# Estimating thermal death time curves in *Drosophila* *tripunctata* using infrared activity monitors

**DOI:** 10.17912/micropub.biology.002093

**Published:** 2026-06-08

**Authors:** Aaron R Yilmaz, Logan R Kolaske, Ryan A Martin, Sarah E Diamond

**Affiliations:** 1 Department of Biology, Case Western Reserve University

## Abstract

Assessing heat failure across different test temperatures allows for the estimation of heat tolerance and thermal sensitivity under the thermal death time (TDT) model of damage accumulation. One challenge is phenotyping sufficient numbers of individuals. Previous work used human observations or cameras. Infrared (IR) monitors provide an alternative method. While IR monitors have been successfully used to assess heat failure, they have not been used to estimate TDT parameters. Here, we use *Drosophila tripunctata* to estimate heat tolerance and thermal sensitivity for males and females across 4-6 test temperatures and 6 isofemale lines, validating the use of IR monitors.

**Figure 1. Conceptual framework and empirical thermal death time relationships:** (A) Conceptual expectation (not derived from empirical data) showing the expected decrease in heat knockdown time (HKDT) with increasing static temperature. (B) Conceptual illustration of thermal death time (TDT) parameter estimation on a log-transformed scale, showing calculation of instantaneous CTmax at a 10-minute exposure (CTmax10) and the slope parameter *z* (thermal sensitivity; *z* = -1/slope). (C) Observed HKDT across six static temperatures (35–40° C) for six isolines of *D. tripunctata* . Points represent individual flies (triangle = female, circle = male). Colors distinguish isolines, and lines show fitted trends by isoline (pooled across sex). (D) TDT relationships after log10 transformation of HKDT, with linear fits used to estimate CTmax10 and z for each isoline group (pooled across sex). Points represent individual flies (triangle = female, circle = male). Colors correspond to isolines as in panel C. (E) Observed HKDT for males (dashed line) and females (solid line) across six static temperatures (35–40° C). Points represent individual flies (triangle = female, circle = male). Colors distinguish sex (green = female, purple = male), and lines show fitted trends by sex (pooled across isolines). (F) TDT relationships for males (dashed line) and females (solid line) after log10 transformation of HKDT, with linear fits used to estimate CTmax10 and z for each sex (pooled across isolines). Points represent individual flies (triangle = female, circle = male). Colors and line types correspond to sex as in panel E.

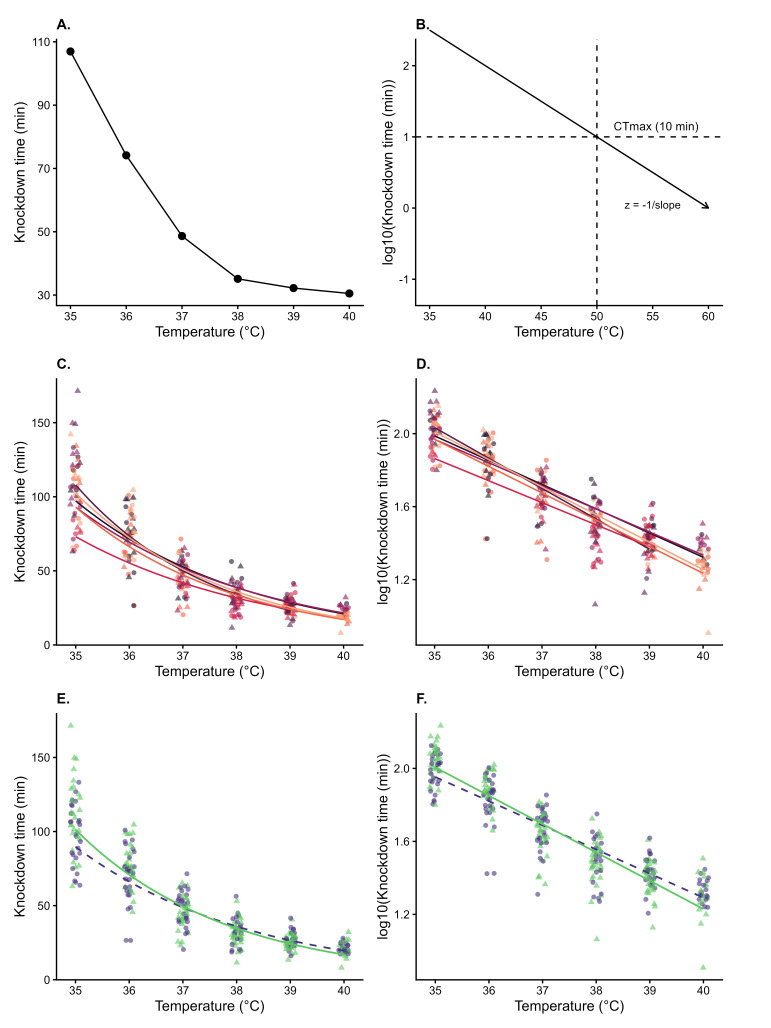



## Description

Thermal performance and tolerance traits are key components of species ecology and evolution, and are vital for use in forecasts of responses to climatic warming (Huey et al., 2012). Thermal death time (TDT) models are a powerful, mechanistic framework for quantifying the accumulation of thermal damage by integrating descriptions of both duration and intensity of exposure to thermal stress (Jørgensen et al., 2021; Ørsted et al., 2022). As a consequence, these models provide estimates of heat tolerance (the critical thermal maximum, CTmax) and thermal sensitivity (the slope of the relationship between the time and temperature at which heat failure occurs). Heat tolerance and thermal sensitivity are biologically meaningful parameters that can be compared across species. 

To collect the data necessary to estimate TDT parameters, prior studies have used water baths with either visual or camera-based scoring to assess the timing of heat failure, often using heat knockdown time (HKDT) defined as the loss of movement, across multiple test temperatures (Alruiz et al., 2022; Castañeda et al., 2015). Infrared (IR) activity monitors could provide an efficient tool for collecting HKDT data as the monitors record continuous measurements over time and accompanying software can automate the estimation of the timing of loss of movement. IR-based activity monitors have been used to compute heat knockdown time (HKDT) at two static test temperatures (Rokusek et al., 2023), but have not been used to collect HKDT for use in TDT parameter estimation. Here, we validate the use of IR monitors for the collection of HKDT data and the use of these data in TDT models to estimate CTmax and thermal sensitivity. We used adult *Drosophila* *tripunctata* from individual isofemale lines established from a mass-reared laboratory population originating with flies captured off mushroom baits as our study population.

We had a total of 6 isofemale lines and 4 to 6 test temperatures, depending on the isoline (3 isolines had 4 test temperatures and 3 isolines had 6 test temperatures). Test temperatures ranged from 35 to 40 °C. For the isolines tested across fewer temperatures, all minimum test temperatures were 35 °C and maximum test temperatures were either 39 or 40 °C. Among these different groups, we tested a total of 299 individual flies, including both males and females. Mean replication per group (each combination of temperature, isoline, and sex) was 4.98, and ranged from 2 to 13 individuals. 

We quantified HKDT as the time (to the resolution of seconds) at which the last movement was recorded, using a 5-minute buffer of no activity to ensure heat knockdown had occurred. We used these HKDT data to create linear TDT models (log-transformed HKDT as a function of temperature) for each isoline-by-sex group. From these TDT models, we obtained both the instantaneous CTmax at a 10-minute exposure (CTmax10) and the thermal sensitivity parameter z (defined as -1/slope). CTmax10 is a commonly used exposure time and was chosen because it reflects an ecologically relevant heat stress duration. Depending on the species and test temperatures, the calculation of CTmax over shorter exposure durations can lead to extrapolation beyond the data range (Jørgensen et al., 2021), i.e. our lowest HKDT was 8.08 minutes. 

We first assessed the overall fits of the TDT models to the HKDT data and then explored differences in modeled TDT parameters between sexes and isolines. Model fits were reasonable across sex-by-isoline groups, with a mean R² value of 0.795 and range of 0.677-0.919 (n = 12 groups). Unsurprisingly, model fits improved with more test temperatures, i.e. the mean R² value of models fit with 6 test temperatures was 0.837 with a range of 0.724-0.919. These R² values are comparable to those reported for TDT curves in other systems (e.g., Jørgensen et al. 2019; Li et al. 2023).

Because sex can strongly influence CTmax and thermal sensitivity (Castañeda et al. 2015), we used linear mixed effects models to explore how sex affected each response, with isoline included as a random effect. We found that sex had a significant effect on both CTmax10 (𝜒 ^2^ = 9.80, p = 0.00175) and thermal sensitivity (𝜒 ^2^ = 12.6, p = 0.000385). Specifically, males had higher CTmax10 than females (males: mean [95% CI] = 42.3 [41.8, 42.8]; females: mean [95% CI] = 41.7 [41.2, 42.3]; contrast females - males ± SE = -0.525 ± 0.168, t = -3.13, p = 0.0259). Males had lower sensitivity (higher z) than females (males: mean [95% CI] = 7.66 [6.84, 8.49]; females: mean [95% CI] = 6.87 [6.05, 7.70]; contrast females - males ± SE = -0.790 ± 0.223, t = -3.55, p = 0.0164). These results are similar to other studies that show evidence of sex biases in CTmax and thermal sensitivity (Castañeda et al. 2015; Pottier et al. 2021; Soto et al. 2025; Vey et al. 2026).

To provide a coarse estimate of genetic variation in CTmax and thermal sensitivity, we examined the variance components for isoline from each mixed model (accounting for sex as a fixed effect). The variance attributable to isoline (after accounting for sex) was 0.687 in the model of CTmax10 and 0.785 in the model of thermal sensitivity. High genetic variance in heat tolerance and thermal sensitivity was also found in a study of *D. melanogaster* (100 lines from the Drosophila Genetic Reference Panel), where the 95% confidence intervals of broad sense heritabilities for CTmax ranged between 0.42 and 0.73, and for thermal sensitivity, ranged between 0.45 and 0.75 (Soto et al. 2025).

To understand how isoline altered the estimation of CTmax10 and thermal sensitivity of *D. tripunctata* , we constructed a new set of models with isoline (and sex) included as fixed effects (rather than random effects). Depending on the isoline, CTmax10 ranged from 41.2 to 42.4 for females and between 41.7 and 42.9 for males. Thermal sensitivity ranged from 5.88 to 7.93 for females and 6.67 to 8.72 for males. In aggregate, these values of CTmax are positively biased compared with our previous estimates of CTmax in this system (Diamond et al. 2022), where CTmax was estimated as 37.3 to 38.7 for females and 36.7 to 38.1 for males (depending on population of origin) using a dynamic temperature ramping protocol. This discrepancy could be driven by a number of different factors including differences in exposure to heat stress between the static versus dynamic assessments of CTmax, differences in observation-based versus IR-based assessments of heat failure, differences arising from sampling biases of wild populations, or differences in the number of generations of laboratory rearing and rearing temperatures (22 vs 20 °C in Diamond et al. 2022) between these two studies.

Further, the higher CTmax10 of males compared with females in our study was unexpected given previous findings from this system showing the opposite relationship when CTmax was quantified using a dynamic temperature ramping protocol (Diamond et al. 2022). It is possible that females are better than males at coping with the specific challenges of the dynamic ramping design–increases in temperature of 0.5 °C every minute starting at 22 °C–but worse than males when immediately exposed to a stressfully high temperature. Other studies in *Drosophila* have detected similar patterns. For example, Castañeda et al. (2015) and Soto et al. (2025) found higher CTmax in males ( *D. subobscura* and *D. melanogaster* , respectively) compared with females when estimating CTmax from TDT curves based on heat knockdown time under static temperature treatments. However, given the many other differences besides the static versus ramping design between our study and previous work in *D. tripunctata* , it is unclear whether the different patterns of sex-dependence in CTmax represent biologically meaningful variation in how males and females respond to temperature stress or whether these differences represent unintentional methodological variation among studies such as biases in population sampling. Regardless, the dependence of both CTmax and thermal sensitivity on sex in our study reinforces the utility of quantifying both components of heat failure.

Finally, we drew comparisons across our two response variables, CTmax and thermal sensitivity, to explore the potential for positive or negative covariance, as expected by different models of evolution of thermal physiology (Ørsted et al. 2022). We modeled CTmax10 as a function of z, with random effects for sex and isoline and found a significant effect of z (𝜒 ^2^ = 75.3, p < 0.0001) such that higher z values (lower sensitivity) were associated with higher CTmax10 (mean [95% CI] = 0.636 [0.453, 0.778]). This result suggests a tradeoff between heat tolerance and thermal sensitivity over the range of temperatures in our experiment, though we emphasize caution here with how broadly these results might hold given the low statistical power.

In sum, our study shows that IR monitors can be used to assess HKDT, and that when such data are used to parameterize TDT models, the resulting estimates of CTmax and thermal sensitivity are broadly comparable to those of other studies. Along with currently used observation-based and camera-based methods for assessing HKDT and estimating TDT parameters (e.g., Pérez-Gálvez et al. 2023; Soto et al. 2025), IR monitors can be considered as another tool in the toolbox of assessing components of heat failure.

## Methods

*Fly Rearing*

The *Drosophila tripunctata* used in this study were from a mass-reared lab population with overlapping generations that originated from isolines of wild-caught females collected in the fall of 2024 from mushroom baits at 3 forested sites in Cuyahoga and Geauga counties, Ohio (latitude, longitude [41.495043, -81.408543; 41.491499, -81.408131; 41.510243, -81.357476]). The mass-reared population was founded by a total of 462 flies (~ 50:50 sex ratio), originating from 9 separate isofemale lines in November, 2024. This mass-reared population was reared on Formula 4-24 Instant Drosophila Medium (Carolina Biological Supply) and kept at 18 °C under a 12L:12D light cycle (PHCBI MIR-154-PA) for approximately 12 months before creating isolines used in this study. We established 6 isofemale lines from the mass-reared population which were likewise fed Formula 4-24 Instant Drosophila Medium (Carolina Biological Supply) but incubated at 22 °C on a 12L:12D light cycle (PHCBI MIR-154-PA).

*Heat Knockdown Time Assessments*

We tested the offspring of the isofemale lines 2-3 days post-eclosion at 6 static temperatures (35, 36, 37, 38, 39, and 40°C). All testing was performed in growth chambers (Percival Scientific 36-VL). Individual flies were anesthetized with CO₂ for up to 8 minutes before being placed in 5 mm x 65 mm glass test tubes with a black plastic cap at one end, and a small piece of cotton ball at the other end. The flies were then allowed at least 15 minutes to recover before they were loaded into the IR sensor array. Heat knockdown time (HKDT) was assessed using TriKinetics DAM5H infrared activity monitors (Princeton, MA), and HKDT was defined as the last registered movement made by an individual followed by at least 5 minutes of no recorded activity. We used the coding scripts provided in Rokusek et al. 2023 to automatically detect the timing of the last movement made for each individual in the experiment.

*Statistics*

Statistical analyses were performed using R version 4.5.2 (R Core Team, 2023). We log₁₀-transformed HKDT prior to analysis. For each isoline-by-sex group, we fit TDT curves using linear regression models of log₁₀(HKDT) as a function of temperature (using “lm” from the base R package). We assessed model fit using the coefficient of determination (R²). We extracted both the instantaneous CTmax at a 10-minute exposure (CTmax10) and the thermal sensitivity parameter z (calculated as -1/slope) from each fitted model. 

We tested for differences in CTmax10 and z among sexes and isolines using linear mixed effects modeling with the *lmer* function from the lme4 package (Bates et al. 2015). We used *Anova* from the car package (Fox and Weisberg 2019) to assess the significance of predictors. We used the *emmeans* function and package (Lenth 2019) to estimate the modeled group means and their associated errors.

*Data Availability*

Data are available at the Open Science Framework, https://doi.org/10.17605/OSF.IO/Y249J
